# Comparative Genomics of Closely-Related *Gordonia* Cluster DR Bacteriophages

**DOI:** 10.3390/v14081647

**Published:** 2022-07-27

**Authors:** Cyril J. Versoza, Abigail A. Howell, Tanya Aftab, Madison Blanco, Akarshi Brar, Elaine Chaffee, Nicholas Howell, Willow Leach, Jackelyn Lobatos, Michael Luca, Meghna Maddineni, Ruchira Mirji, Corinne Mitra, Maria Strasser, Saige Munig, Zeel Patel, Minerva So, Makena Sy, Sarah Weiss, Susanne P. Pfeifer

**Affiliations:** 1 Center for Evolution and Medicine, School of Life Sciences, Arizona State University, Tempe, AZ 85281, USA; cversoza@asu.edu; 2 Biodesign Institute, School of Life Sciences, Arizona State University, Tempe, AZ 85281, USA; aahowel3@asu.edu; 3 School of Life Sciences, Arizona State University, Tempe, AZ 85281, USA; taftab@asu.edu (T.A.); mmblanco@asu.edu (M.B.); akbrar@asu.edu (A.B.); emchaffe@asu.edu (E.C.); nwhowell@asu.edu (N.H.); jlobatos@asu.edu (J.L.); mluca@asu.edu (M.L.); rmirji@asu.edu (R.M.); cmitra@asu.edu (C.M.); mbstrass@asu.edu (M.S.); smunig@asu.edu (S.M.); zspatel1@asu.edu (Z.P.); minervas@asu.edu (M.S.); mrsy2@asu.edu (M.S.); sweiss13@asu.edu (S.W.); 4 School of Mathematical and Statistical Sciences, Arizona State University, Tempe, AZ 85281, USA; waleach@asu.edu; 5 School of Molecular Sciences, Arizona State University, Tempe, AZ 85281, USA; mmaddine@asu.edu

**Keywords:** bacteriophage, cluster DR, *Gordonia*, comparative genomics, host range

## Abstract

Bacteriophages infecting bacteria of the genus *Gordonia* have increasingly gained interest in the scientific community for their diverse applications in agriculture, biotechnology, and medicine, ranging from biocontrol agents in wastewater management to the treatment of opportunistic pathogens in pulmonary disease patients. However, due to the time and costs associated with experimental isolation and cultivation, host ranges for many bacteriophages remain poorly characterized, hindering a more efficient usage of bacteriophages in these areas. Here, we perform a series of computational genomic inferences to predict the putative host ranges of all *Gordonia* cluster DR bacteriophages known to date. Our analyses suggest that BiggityBass (as well as several of its close relatives) is likely able to infect host bacteria from a wide range of genera—from *Gordonia* to *Nocardia* to *Rhodococcus,* making it a suitable candidate for future phage therapy and wastewater treatment strategies.

## 1. Introduction

Bacteriophages are one of the most abundant organisms on Earth, infecting a wide range of host bacteria present in almost any environment from common garden soil to volcanic substrates and from freshwater streams to oceans [1]. Among these hosts, members of the order *Corynebacteriales*—including *Gordonia*, *Mycobacterium*, *Nocardia*, and *Rhodococcus*—are of particular importance to agriculture, biotechnology, and medicine as the outer membrane of their bacterial cells, which consists of long-chain hydroxylated mycolic acids, frequently leads to complications during the prevention, treatment, and cure of opportunistic pathogens [2]. Moreover, due to the hydrophobic nature of this “mycomembrane”, *Corynebacteriales* often cause severe problems during wastewater treatment as they can stabilize foams on the surface of aeration tanks during the activated sludge phase [3], which not only complicates sludge management and increases maintenance costs but also poses a health hazard to wastewater treatment plant workers in their aerosolized form [4].

Owing to the growing scarcity of clean water across the globe, treated wastewater serves as an important alternative to freshwater for many nations with more than 35% of agricultural irrigation, 17% of landscape irrigation, and 12% of groundwater recharge in the United States stemming from treated wastewater [5]. However, microbial hazards, such as multi-drug resistant bacterial pathogens, are frequently discharged into sewage systems due to the common usage of antibiotics in animal farms and on crop fields. Consequently, effective wastewater treatment strategies are indispensable to combat environmental and health concerns for farmers and consumers alike [6].

Due to their host specificity, lytic bacteriophages have been proposed as promising and environmentally-friendly bacterial treatment and control agents to remove harmful (or otherwise problematic) bacteria—such as gram-positive *Gordonia* which are associated with both systemic infections in immunocompromised and local infections in immunocompetent individuals [7,8] as well as sludge foaming [9,10]—while maintaining desirable microorganisms in the wastewater. To effectively guide these biological control strategies, bacteriophages and their host ranges (i.e., the bacterial genera and species a bacteriophage is able to infect) must be well-characterized—yet, the diversity of *Gordonia* bacteriophages remains largely unexplored.

As part of a course-based undergraduate research experience at Arizona State University, we computationally inferred putative host ranges of all *Gordonia* cluster DR bacteriophages known to date to aid the design and improvement of future wastewater treatment strategies.

## 2. Materials and Methods

Genomic data for *Gordonia* cluster DR bacteriophages (Supplementary Table S1) were explored using Phamerator [11] and phylogenetic relationships characterized together with representative *Microbacterium*, *Mycobacterium*, and *Streptomyces* bacteriophages as outgroups (Supplementary Table S2). Specifically, MAFFT v.7 [12] embedded within the EMBL-EBI Bioinformatics Toolkit [13,14] was used to generate a multiple-sequence alignment between the bacteriophages. The resulting alignment was then used to generate a neighbor-joining tree in MEGA X [15] using a phylogeny test with 10,000 bootstrap replicates. Nucleotide sequence relatedness was assessed using Gepard v.2.1.0 [16]. Pairwise average nucleotide identities (ANIs) were calculated using the “Genome Comparison” tool embedded within DNA Master v.5.23.6 and plotted using the ggplot2 package [17] in R v.4.1.0.

Following suggested best practices by Versoza and Pfeifer [18], a combination of exploratory and confirmatory methods was utilized to computationally predict host ranges of the closely-related *Gordonia* cluster DR bacteriophages. Specifically, putative host ranges were predicted using two machine-learning based prediction tools—CHERRY [19] and PHERI v.0.2 [20]—as well as the alignment-free prediction tool WIsH v.1.1 [21] together with genomic data from ten putative bacterial host species spanning three genera—*Gordonia*, *Nocardia*, *Rhodococcus*, and, as a negative control, *Escherichia* (Supplementary Table S3). All software was executed using default settings.

## 3. Results

To confirm cluster membership, the genomes of *Gordonia* cluster DR bacteriophages were investigated. They show a high level of sequence similarity with the left arm of the genomes mostly encoding well-conserved structural and assembly proteins (including a terminase, portal protein, capsid maturation protein as well as major capsid hexamer and pentamer proteins, a head-to-tail adaptor, tail assembly protein, tape measure protein, minor tail protein subunits, lysin A, lysin B, and several genes responsible for integration into the host). Thereby, the RuvC-like resolvase (Supplementary Figure S1), a Holliday junction resolving enzyme that is a distant relative of the RuvC proteins present in gram-negative bacteria such as *Escherichia coli* [22], is of particular interest. It closely resembles the RuvC-like endonucleases found in select *Siphoviridae* and *Myoviridae* bacteriophages infecting *Streptococcus* and *Lactococcus* hosts [23,24], which may hint at a shared evolutionary history. The right arm of the genomes contains non-structural genes (including an exonuclease, DNA helicase, DNA polymerase, and HNH endonuclease). Notably, several cluster DR bacteriophages exhibit a partial toxin/antitoxin (TA) system (Supplementary Figure S2). Prevalent in many archaea and bacteria, TA systems encode a toxin protein and a corresponding antitoxin in the form of a protein or non-coding RNA that serves as a defense mechanism against invading bacteriophages [25,26]. As bacteriophages co-evolve with their bacterial hosts [27], adaptations to such defense mechanisms are common [28] to allow bacteriophages to inactivate bacteria-encoded toxins [29,30]. Indeed, the TA system of the cluster DR bacteriophages is homologous to the *hicA* TA system frequently present in *Burkholderia pseudomallei*, *E. coli*, and *Pseudomonas aeruginosa* [31,32,33].

To elucidate phylogenetic relationships, comparative analyses were performed between all *Gordonia* cluster DR bacteriophages known to date (Supplementary Table S1). Following Pope and colleagues [34], clustering was based on nucleotide similarity and shared gene content, with bacteriophages sharing at least 35% of genes being grouped into clusters. A neighbor-joining tree confirmed membership in the DR cluster (Supplementary Figure S3a)—an assignment that was further supported by both the dot plot analyses (Supplementary Figure S4) as well as the pairwise average nucleotide identities (Supplementary Figure S5). Interestingly, gene trees of the RuvC-like resolvase (Supplementary Figure S3b) and the *hicA*-like toxin (Supplementary Figure S3c) do not recapitulate the whole genome phylogeny—however, it is unclear whether this is due to inconsistent resampling during bootstrapping caused by the short sequence length [35] or the mosaic architecture of the genome caused by horizontal gene transfer by illegitimate recombination [36,37,38]. Compared to temperate bacteriophages, both gene acquisition and gene loss, in lytic bacteriophages is less well understood [39]. However, there have been previous reports of gene transfers in T4-like and T7-like bacteriophages [40,41], and lytic bacteriophages with large genomes have been suggested to have acquired genes from donor genomes [42].

Due to their bactericidal nature, bacteriophages are frequently used for a variety of agricultural, biotechnological, and medical applications [43]. To effectively guide the usage of bacteriophages in these areas, their host ranges have to first be determined (see discussion in [18]). To investigate the host ranges of the closely related cluster DR bacteriophages, a combination of exploratory and confirmatory prediction tools was utilized together with a dataset of ten putative bacterial host species and *E. coli* as a negative control (Supplementary Table S3). Specifically, the tested host dataset spans the three genera of the *Corynebacteriales* order—*Gordonia*, *Nocardia*, and *Rhodococcus*—that have been implicated in activated sludge foaming in wastewater treatment plants [44].

**Figure 1:**
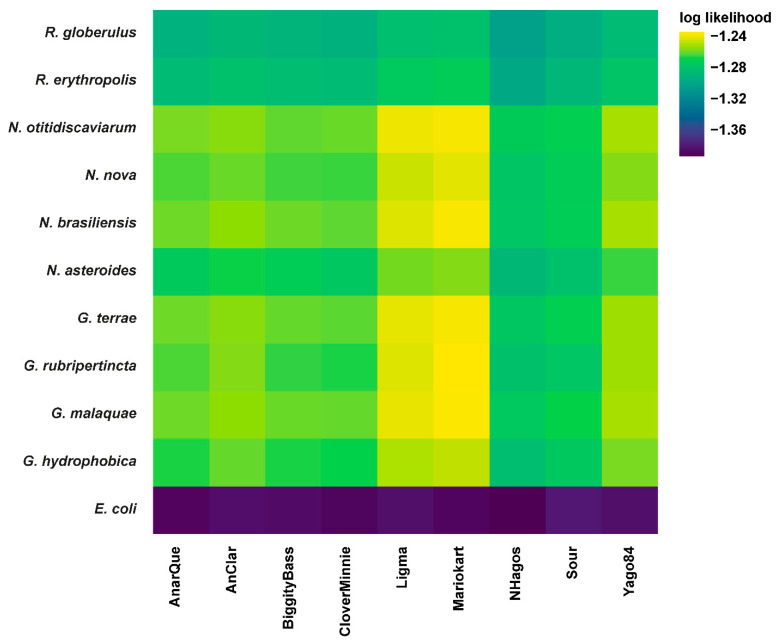
Putative host ranges as predicted by WIsH. Heatmap of log-likelihoods of bacteriophage-host pairs—including nine *Gordonia* cluster DR bacteriophages (Supplementary Table S1) as well as ten potential bacterial hosts and *E. coli* as a negative control (Supplementary Table S3)—generated by the host prediction tool WIsH [21]. Higher values correspond to more likely interactions.

Using the exploratory method PHERI [20], seven out of nine cluster DR bacteriophages were predicted to infect hosts under the *Gordonia* genus (Table 1), with the exception of bacteriophages AnClar and Yago84. To make host range predictions for newly encountered bacteriophages, PHERI utilizes a decision tree classifier of annotated protein clusters of bacteriophages with known hosts. Consequently, bacteriophages will only be predicted to infect a particular host if their protein profile closely matches that of another bacteriophage known to infect that host. As minor tail proteins play an essential role in bacteriophage infection [45], the lack of similarity in the minor tail protein profiles of AnClar and Yago84 compared to those bacteriophages known to infect *Gordonia* hosts might explain why neither were predicted to infect the *Gordonia* genus, despite having been isolated in *G. terrae* (Supplementary Table S1). In fact, the clades observed within the gene tree of the minor tail protein shared across all cluster DR bacteriophages (Supplementary Figure S3d) reflects the clustering of the bacteriophages with respect to host range, reiterating the importance of tail proteins for host infection. Using the exploratory method CHERRY [19]—a graph convolutional encoder and decoder that relies on a broader range of features including protein organization, sequence similarity, and *k*-mer frequency to predict host ranges—highlights *M. smegmatis*, *G. terrae*, and *R. hoagie* as the three most likely host candidates for all cluster DR bacteriophages (though the latter two scoring predictions fell below the recommended confidence threshold of 0.9). Conversely, the confirmatory method WIsH [21]—based on a Markov model that determines the *k*-mer similarity between bacteriophage and host genomes—predicted *G. hydrophobica*, *G. malaquae*, *G. rubripertincta*, and *G. terrae* as potential hosts for all nine cluster DR bacteriophages relative to the negative control, *E. coli* (Figure 1). Moreover, log likelihood values for putative *Nocardia* and *Rhodococcus* hosts were comparable to those of *Gordonia*, suggesting the potential for a much broader host range. Interestingly, BiggityBass exhibits the broadest predicted host range among all cluster DR bacteriophages, spread across five different phyla (Table 1), making it an appealing agent to explore for future wastewater treatment strategies [46].

In conclusion, computational methods can offer a first glimpse into the putative host ranges of newly discovered bacteriophages—yet, it is important to remember that these methods are predictive by their very nature. Thereby, each computational method exhibits their own advantages and limitations. For example, tools that rely solely on *k*-mer-based models can lead to an overprediction of host ranges if convergent evolution resulted in similar nucleotide frequency patterns [47], whereas tools that rely on machine-learning are inherently limited in their predictions by the bacteriophage-host datasets available for training [18]. Experimental validation through bacteriophage isolation and cultivation still remains the “gold standard” in determining bacteriophage host ranges—however, it certainly is not without its own limitations as not all microbial hosts are amendable to cultivation in the laboratory and, even if they are, results may depend on the conditions under which the experiments were performed [18]. Given the ever growing knowledge of bacteriophage diversity across the globe, it is our hope that future computational and experimental research will go hand in hand to further explore polyvalent bacteriophages as an interesting study system to gain a better understanding of the molecular and genetic determinants underlying host range.

## Figures and Tables

**Table 1:** Putative host ranges as predicted by PHERI. Putative hosts of the nine *Gordonia* cluster DR bacteriophages included in this study (Supplementary Table S1) predicted by PHERI [20].

	*Gordonia*	*Arthrobacter*	*Aeromonas*	*Staphylococcus*	*Shigella*	*Corynebacterium*	*Stenotrophomonas*
AnarQue	√	√	√				
AnClar		√	√				
BiggityBass	√	√	√	√	√		
CloverMinnie	√	√	√				
Ligma	√	√	√			√	
Mariokart	√	√	√				
NHagos	√	√	√				
Sour	√	√	√				√
Yago84		√	√				

## Data Availability

Genomic data for *Gordonia* cluster DR bacteriophages, bacteriophages included as outgroups, and putative bacterial host species can be downloaded from the NCBI Sequence Read Archive using the accession numbers provided in Supplementary Tables S1, S2, and S3, respectively.

## Supplementary Materials

The following supporting information can be downloaded at: https://www.mdpi.com/article/10.3390/v14081647/s1, Figure S1: Phamerator map of the RuvC-like resolvase gene; Figure S2: Phamerator map of the *hicA*-like toxin gene; Figure S3: Neighbor-joining trees; Figure S4: Dot plots; Figure S5: Average nucleotide identities; Table S1: *Gordonia* cluster DR bacteriophages included in the comparative analyses; Table S2: Bacteriophages included as outgroups in the comparative analyses; Table S3: Host bacteria included in the comparative analyses [48,49,50,51,52,53,54,55,56,57,58].
