# Supplementary material for: Comparative Genomics of Closely-Related Gordonia Cluster DR Bacteriophages
Source: Viruses. 2022 Jul 27;14(8):1647. doi: 10.3390/v14081647 (PMC9413003; doi:10.3390/v14081647)
Supplement: Supplementary file 1 [file viruses-14-01647-s001.zip › Versoza Howell etal - Comparative genomics of closely-related Gordonia cluster DR bacteriophages - Supplementary Figures - CLEAN.pdf]

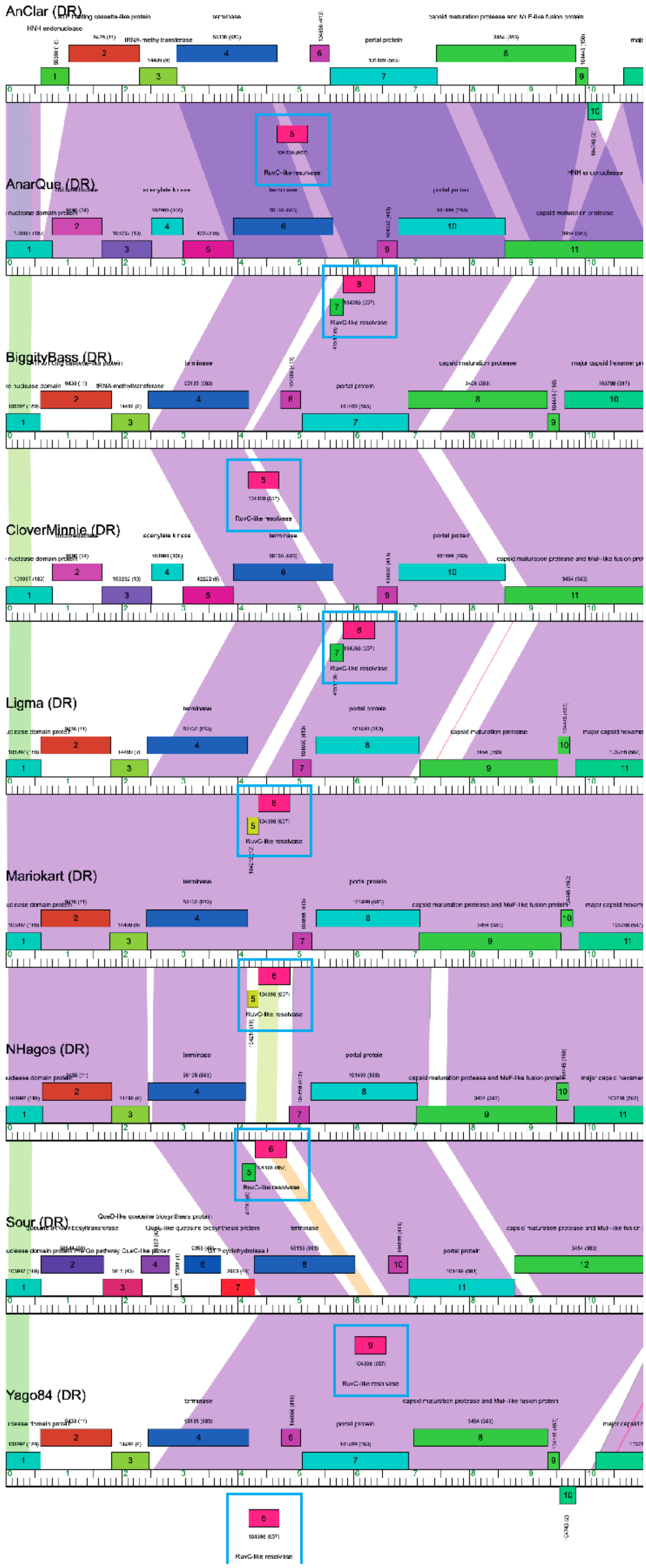

Supplementary Figure S1. Phamerator map of the RuvC-like resolvase gene of closely related *Gordonia* cluster DR bacteriophages (Supplementary Table S1). In this Phamerator map, protein-coding genes with their putative functional assignments (if available) are displayed above or below a ruler, signifying genes on forward or reverse strands, respectively. The numbers shown above each gene indicate the protein family (pham) and, in parenthesis, the number of members in the pham family. Coloring between genomes represents nucleotide similarity with areas of highest similarity shown in purple (BLAST e-value = 0), followed by red (BLAST e-value of ~10-4) and white (no significant similarity).

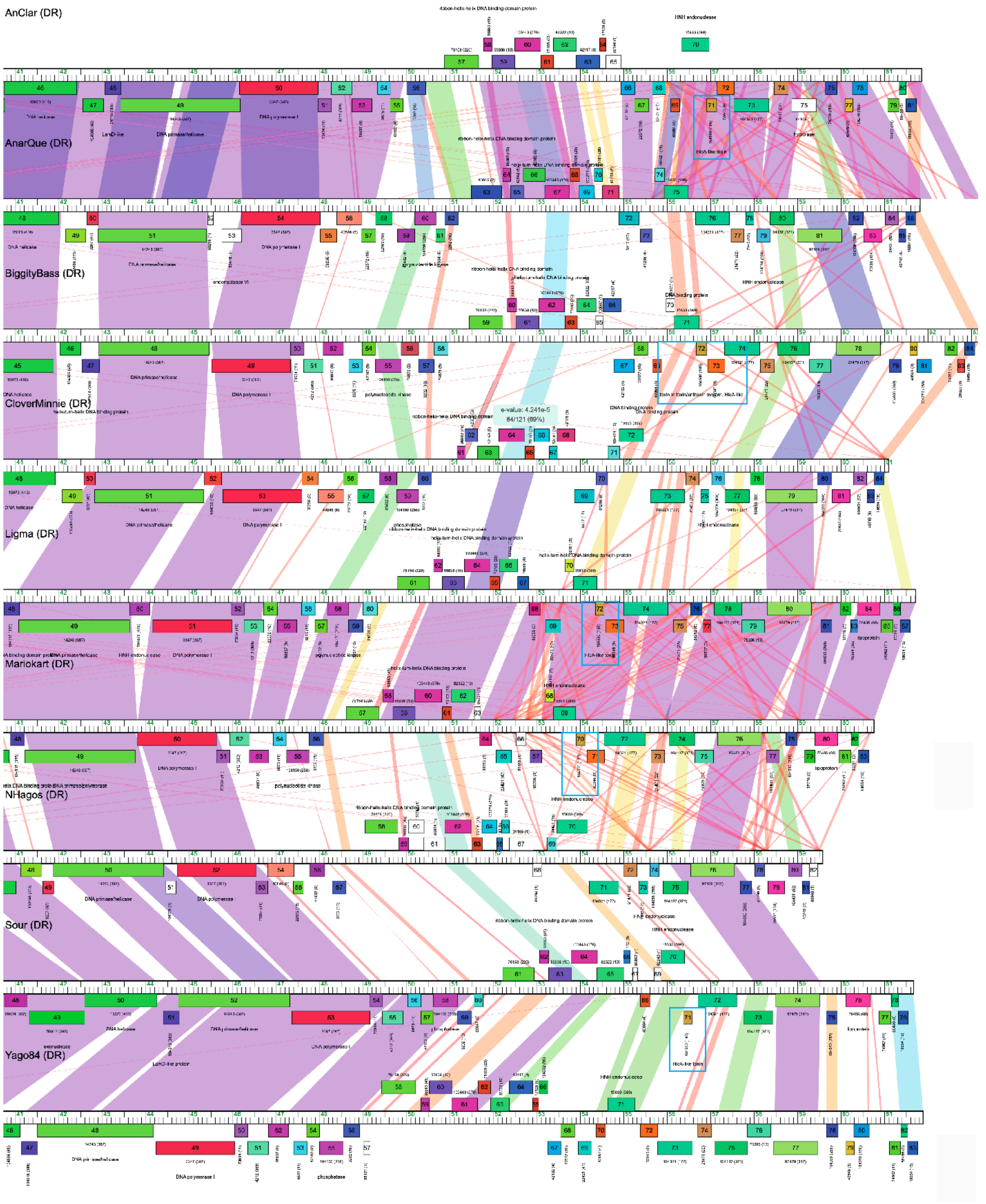

Supplementary Figure S2. Phamerator map of the *hicA*-like toxin gene of closely related *Gordonia* cluster DR bacteriophages (Supplementary Table S1). In this Phamerator map, protein-coding genes with their putative functional assignments (if available) are displayed above or below a ruler, signifying genes on forward or reverse strands, respectively. The numbers shown above each gene indicate the protein family (pham) and, in parenthesis, the number of members in the pham family. Coloring between genomes represents nucleotide similarity with areas of highest similarity shown in purple (BLAST e-value = 0), followed by red (BLAST e-value of ~10-4) and white (no significant similarity).

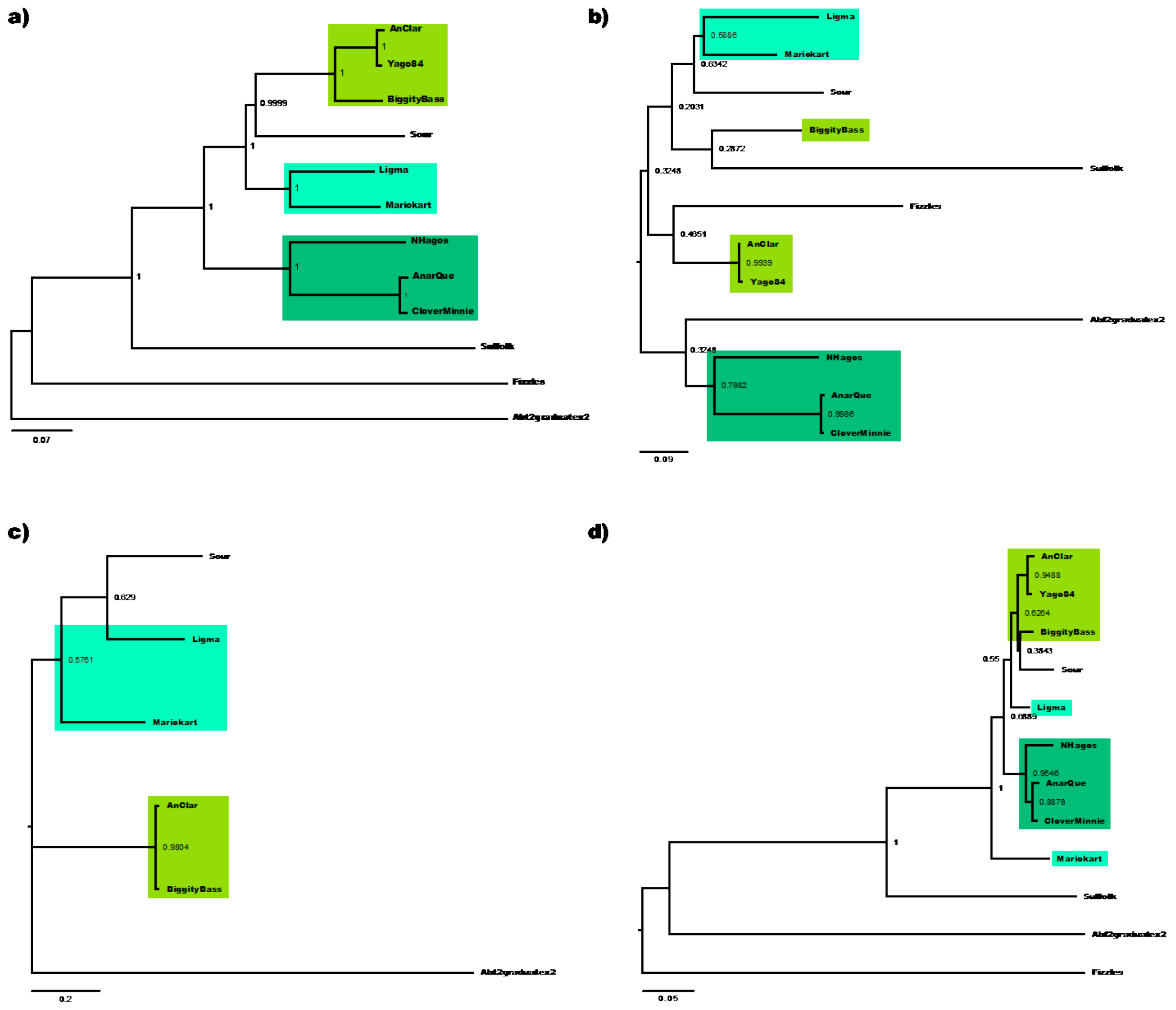

Supplementary Figure S3. Neighbor-joining trees generated in MAFFT using the multiple-sequence alignment of (a) nine *Gordonia* cluster DR bacteriophage genomes (Supplementary Table S1) and their corresponding (b) RuvC-like resolvase, (c) *hicA*-like toxin gene, and (d) minor tail protein with 10,000 bootstrap replicates. Representative *Microbacterium*, *Mycobacterium*, and *Streptomyces* bacteriophages were included as outgroups (Supplementary Table S2).

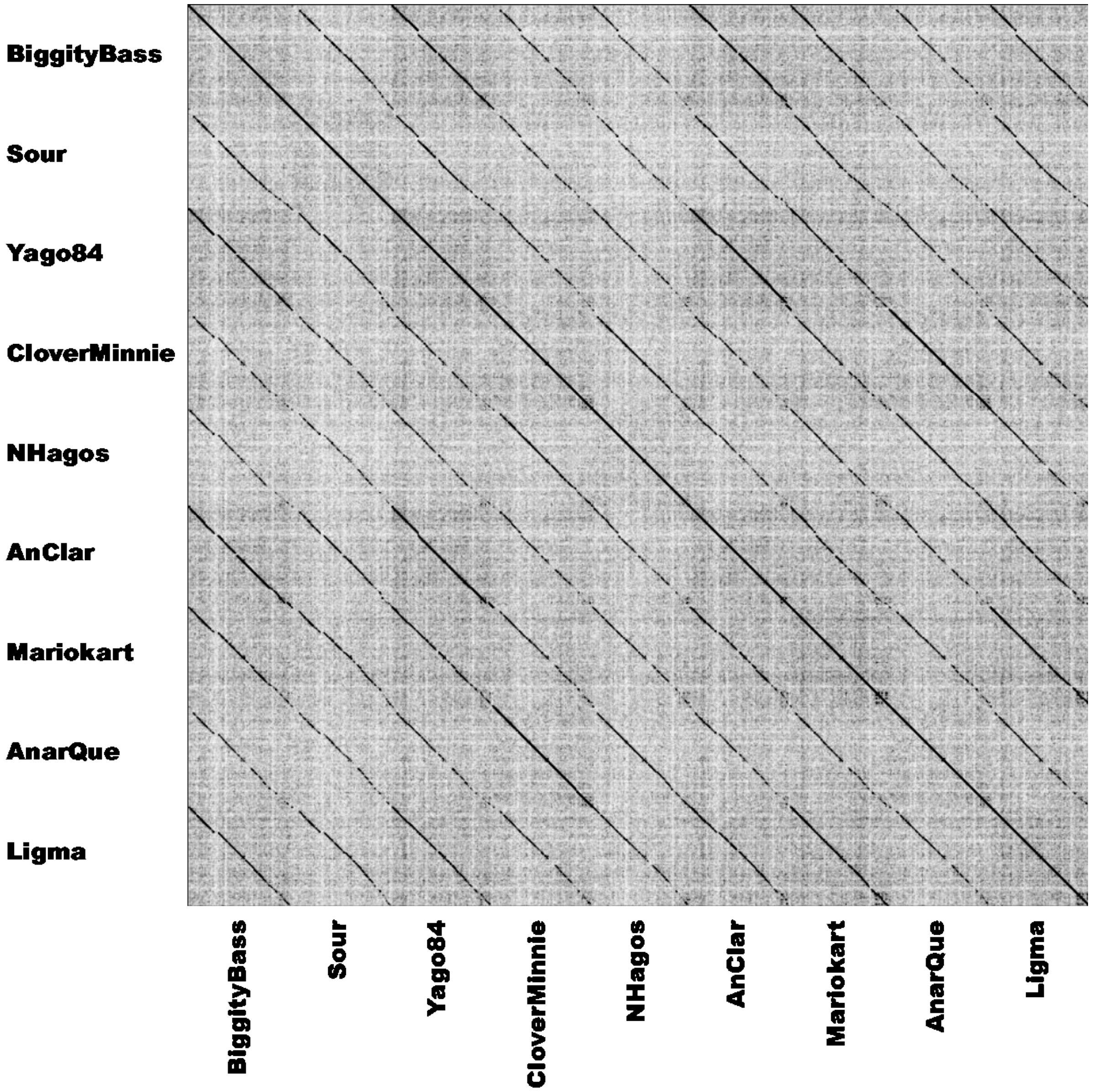

Supplementary Figure S4. Dot plots of closely-related *Gordonia* cluster DR bacteriophages (Supplementary Table S1).

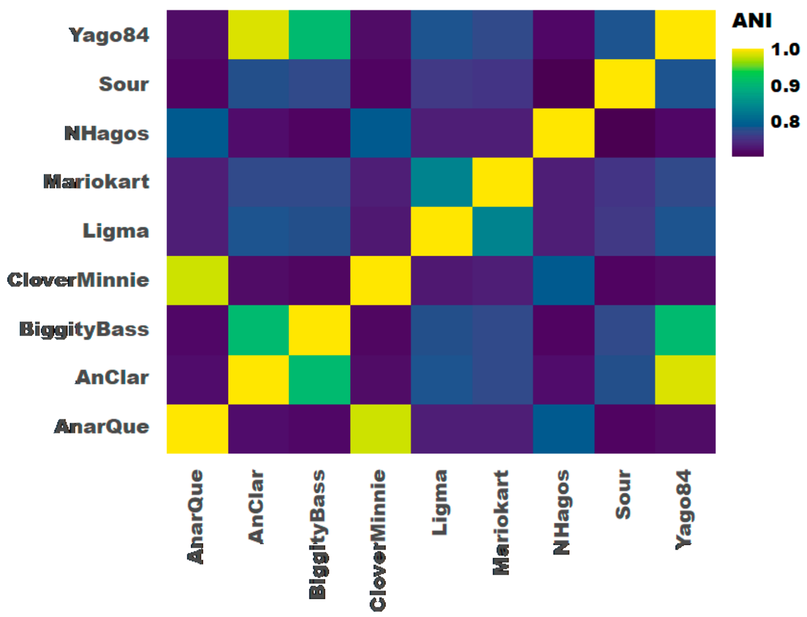

Supplementary Figure S5. Average nucleotide identities (ANIs) of closely-related *Gordonia* cluster DR bacteriophages (Supplementary Table S1).
