# Supplementary material for: Comparative Genomics of Closely-Related Gordonia Cluster DR Bacteriophages
Source: Viruses. 2022 Jul 27;14(8):1647. doi: 10.3390/v14081647 (PMC9413003; doi:10.3390/v14081647)
Supplement: Supplementary file 1 [file viruses-14-01647-s001.zip › Versoza Howell etal - Comparative genomics of closely-related Gordonia cluster DR bacteriophages - Supplementary Tables - CLEAN.pdf]

Supplementary Table S1. *Gordonia* cluster DR bacteriophages included in the comparative analyses. For detailed information on each bacteriophage, please visit the Howard Hughes Medical Institute (HHMI) – Science Education Alliance (SEA) Phage Hunters Advancing Genomics and Evolutionary Science (PHAGES) website at http://phagesdb.org.

Phage Name	Isolation host	Length (bp)	GC-content	# ORFs	# tRNAs	Accession #	Reference
AnarQue	*G. rubripertincta* NRRL B-16540	61,822	68.8	86	0	OK216879	Curran *et al.* 2022
AnClar	*G. terrae* 3612	61,856	69.8	81	1	MN908693	unpublished
BiggityBass	*G. terrae* CAG3	63,202	69.4	83	0	ON260813	Versoza, Howell *et al.*
CloverMinnie	*G. terrae* 3612	61,098	68.7	84	0	MN234196	unpublished
Ligma	*G. terrae* NRRL B-16283	61,714	70.2	87	0	OM105886	unpublished
Mariokart	*G. terrae* NRRL B-16283	60,762	70.5	83	0	MT657335.1	unpublished
NHagos	*G. rubripertincta* NRRL B-16540	59,580	68.2	82	0	MN369758.1	Harrington *et al.* 2020
Sour	*G. terrae* NRRL B-16283	61,670	68.0	79	1	NC_042132.1	unpublished
Yago84	*G. terrae* 3612	61,890	70.0	83	0	MK801725.1	Pope *et al.* 2020

Supplementary Table S2. Bacteriophages included as outgroups in the comparative analyses. Abt2graduatex2 contains a *hicA*-like toxin (pham 34446) whereas Fizzles and Suffolk contain a RuvC-like resolvase (pham 34304). For detailed information on each bacteriophage, please visit the Howard Hughes Medical Institute (HHMI) – Science Education Alliance (SEA) Phage Hunters Advancing Genomics and Evolutionary Science (PHAGES) website at http://phagesdb.org.

Phage Name	Isolation host	Length (bp)	GC-content	# ORFs	# tRNAs	Accession #	Reference
Abt2graduatex2	*S. griseus* ATCC 10137	57,385	69.2	71	0	MF975638.1	Erill & Caruso 2018
Fizzles	*M. foliorum* NRRL B-24224	62,078	68.2	104	0	MW924638.1	unpublished
Suffolk	*M. smegmatis* mc^2 155	68,262	66.6	97	0	KF713485.1	Pope *et al.* 2015

Supplementary Table S3. Host bacteria included in the comparative analyses.

Host	# Genes	Length (kb)	GC-content	Accession #	Reference
E. coli	4,091	4,509	50.6	CP028765	Kang *et al.* 2021
G. hydrophobica	3,962	4,579	67.5	JAFBGB010000001	unpublished
G. malaque	4,349	4,714	66.2	FNRZ01000008	unpublished
G. rubripertincta	4,740	5,104	67.5	CP059694	Han *et al.* 2020
G. terrae	4,979	5,709	67.8	CP029604	unpublished
N. asteroides	6,341	6,987	71.8	CP089214	Sichtig *et al.* 2019
N. brasiliensis	7,949	8,936	68.2	CP022088	Sichtig *et al.* 2019
N. nova	7,450	8,349	67.8	CP006850	Luo *et al.* 2014
N. otitidiscaviarum	6,735	7,688	69.1	CP041695	unpublished
R. erythropolis	6,025	6,509	62.5	CP032403	unpublished
R. globerulus	6,013	6,740	61.7	CP079698	Lozano-Andrade *et al.* 2021

REFERENCES

Curran, E., S. E. Callaway, R. R. Dumanlang, A. V. Harshaw, P. N. Palacio, et al. 2022. Genome sequences of *Gordonia rubripertincta* bacteriophages AnarQue and Figliar. *Microbiol. Resourc. Announc*. 11(1): e01085-21.

Erill, I., S. M. Caruso. 2018. Complete genome sequence of *Streptomyces* bacteriophage abt2graduateex2. *Genome Announc*. 6(3): e01480-17.

Han, S. S., H. K. Kang, B. Y. Jo, B. G. Ryu, H. M. Jin, et al. 2020. Complete genome sequence of *Gordonia rubripertincta* SD5, a soil bacterium isolated from a Di-(2-Ethylhexyl) Phthalate-degrading enrichment culture. *Microbiol. Resourc. Announc*. 9(45): e01087-20.

Harrington, D. A. L., J. L. Stevens, M. J. Johnson, S. J. Pochiro, M. M. Moriarty, et al. 2020. Genome sequences of *Gordonia rubripertincta* bacteriophages Jellybones and NHagos. *Microbiol. Resourc. Announc*. 9(40): e00935-20.

Kang, Y., L. Yuan, X. Shi, Y. Chu, Z. He, et al. 2021. A fine-scale map of genome-wide recombination in divergent *Escherichia coli* population. *Brief. Bioinform*. 22(4): bbaa335.

Lozano-Andrade, C. N., M. L. Strube, Á. T. Kovács. 2021. Complete genome sequences of four soil-derived isolates for studying synthetic bacterial community assembly. *Microbiol. Resourc. Announc*. 10(46): e00848-21.

Luo, Q., S. Hiessl, A. Poehlein, R. Daniel, A. Steinbüchel. 2014. Insights into the microbial degradation of rubber and gutta-percha by analysis of the complete genome of *Nocardia nova* SH22a. *Appl. Environ. Microbiol*. 80(13): 3895–3907.

Pope, W. H., C. A. Bowman, D. A. Russell, D. Jacobs-Sera, D. J. Asai, et al. 2015. Whole genome comparison of a large collection of mycobacteriophages reveals a continuum of phage genetic diversity. *Elife*. 4: e06416.

Pope, W. H., K. A. Butela, R. A. Garlena, D. Jacobs-Sera, D. A. Russell, et al. 2020. Genome sequences of 20 bacteriophages isolated on *Gordonia terrae*. *Microbiol. Resourc. Announc*. 9(3): e01489-19.

Sichtig, H., T. Minogue, Y. Yan, C. Stefan, A. Hall, et al. 2019 FDA-ARGOS is a database with public quality-controlled reference genomes for diagnostic use and regulatory science. *Nat. Commun*. 10(1), 1–13.

Versoza, C.J., A.A. Howell, T. Aftab, M. Blanco, A. Brar, E. Chaffee, N. Howell, W. Leach, J. Lobatos, M. Luca, et al. The complete genome sequence of the *Gordonia* bacteriophage BiggityBass. Accepted, *Microbiol. Resourc. Announc*.
